# Towards a Formal Genealogical Classification of the Lezgian Languages (North Caucasus): Testing Various Phylogenetic Methods on Lexical Data

**DOI:** 10.1371/journal.pone.0116950

**Published:** 2015-02-26

**Authors:** Alexei Kassian

**Affiliations:** 1 Department of Anatolian and Celtic Languages, Institute of Linguistics of the Russian Academy of Sciences, Moscow, Russia; 2 School for Advanced Studies in the Humanities, Russian Presidential Academy of National Economy and Public Administration, Moscow, Russia; Swiss Federal Institute of Technology (ETH Zurich), SWITZERLAND

## Abstract

A lexicostatistical classification is proposed for 20 languages and dialects of the Lezgian group of the North Caucasian family, based on meticulously compiled 110-item wordlists, published as part of the *Global Lexicostatistical Database* project. The lexical data have been subsequently analyzed with the aid of the principal phylogenetic methods, both distance-based and character-based: Starling neighbor joining (StarlingNJ), Neighbor joining (NJ), Unweighted pair group method with arithmetic mean (UPGMA), Bayesian Markov chain Monte Carlo (MCMC), Unweighted maximum parsimony (UMP). Cognation indexes within the input matrix were marked by two different algorithms: traditional etymological approach and phonetic similarity, i.e., the automatic method of consonant classes (Levenshtein distances). Due to certain reasons (first of all, high lexicographic quality of the wordlists and a consensus about the Lezgian phylogeny among Caucasologists), the Lezgian database is a perfect testing area for appraisal of phylogenetic methods. For the etymology-based input matrix, all the phylogenetic methods, with the possible exception of UMP, have yielded trees that are sufficiently compatible with each other to generate a consensus phylogenetic tree of the Lezgian lects. The obtained consensus tree agrees with the traditional expert classification as well as some of the previously proposed formal classifications of this linguistic group. Contrary to theoretical expectations, the UMP method has suggested the least plausible tree of all. In the case of the phonetic similarity-based input matrix, the distance-based methods (StarlingNJ, NJ, UPGMA) have produced the trees that are rather close to the consensus etymology-based tree and the traditional expert classification, whereas the character-based methods (Bayesian MCMC, UMP) have yielded less likely topologies.

## Data

**Fig 1:**
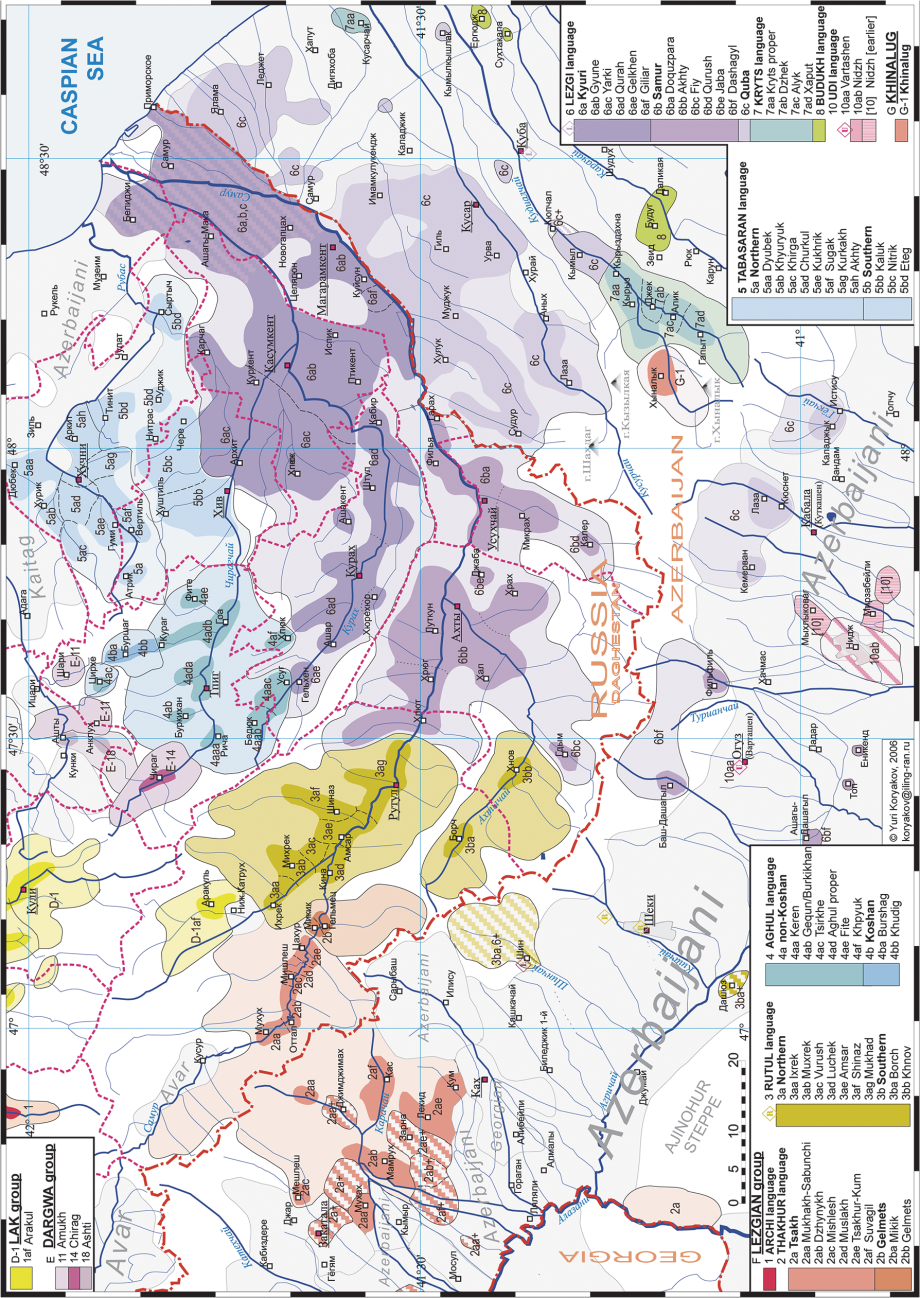
Map of the modern Lezgian lects (adapted from [1]).

Lezgian is a relatively deep linguistic group (deeper than the German, Slavic or Turkic groups, but younger than the Indo-European family) which consists of languages spoken in South-East Dagestan (Russian Federation) and the adjacent parts of Azerbaijan, see Fig. 1 for the geographic map (adapted from [1]). The Lezgian group is a member of the Nakh-Dagestanian cluster of the North Caucasian linguistic family. The traditional expert view is that the Lezgian group consists of two outliers (Udi and Archi) and a large group of nuclear a.k.a. Samur lects which divide into three clusters: West, South and East, see Sec. 4 for detail.

Within the framework of the *Global Lexicostatistical Database* project [2], 110-item wordlists of basic vocabulary for 20 Lezgian languages and dialects have been compiled and annotated by the author [3]. This is the maximum number of Lezgian lects for which available lexicographic sources permit to compile a Swadesh wordlist without field work. The following languages are included in the current version of the GLD Lezgian database: Udi (2 dialects), Archi, Kryts (2 dialects), Budukh, Tsakhur (3 dialects), Rutul (3 dialects), Aghul (5 dialects), Tabasaran (2 dialects), Lezgi. These 20 synchronic wordlists meet the high standards of the *Global Lexicostatistical Database* project.

1. Lexical slots are filled in accordance with the semantic specification of the Swadesh items [4]. Note that despite the fact that the Swadesh wordlist is extensively used by linguists from the mid-20^th^ century, the only attempt to clarify the exact meanings of individual Swadesh items and propose semantic specifications for them was published in 2010. Currently only the *Global Lexicostatistical Database* project consistently adheres to the explicitly stated semantic standard.
2. All relevant sources—dictionaries, grammars, text corpora—are taken into account. This includes not only modern publications, but also data collected and published by Peter von Uslar, Adolf Dirr, Adalbert Starchevsky and other Caucasologists in the late 19^th^—early 20^th^ century.
3. All the analyzed forms are uniformly transcribed in an IPA-based alphabet; additionally, traditional Cyrillic spellings are quoted in parentheses.
4. Lexical lists are annotated: all forms are supplemented with references to the sources and many important phonetic, morphological and semantic details are explicitly discussed in the annotations. This especially concerns occasional (quasi-)synonymy and regular quasi-synonymy. For example, notes on the entries ‘hand’ and ‘foot’ also include an obligatory discussion of expressions for ‘arm’ and ‘leg’ (similarly ‘fog’ and ‘rain cloud’ in the entry for ‘cloud’; ‘hot’ in the entry for ‘warm’; ‘male’ and ‘husband’ in the entry for ‘man’ and so on). Additionally, in notes, forms of those languages and dialects are quoted, whose lexicographic sources are insufficient for 110-item wordlist compiling (e.g., the Udi list contains the relevant lexical data from the Caucasian Albanian palimpsests).

In the aforementioned wordlists, cognations of individual forms were set up in two ways: etymological and phonetic similarity-based. All the described calculations have been doubled for these two options.

Firstly, cognation indexes were marked with help of **traditional comparative method**. I use the Proto-Lezgian reconstruction by the late Sergei Starostin ([5], [6], [7]) with certain corrections and improvements when necessary. S. Starostin’s work is the only full-fledged Proto-Lezgian reconstruction which has been published so far. At the same time, the German Caucasologist Wolfgang Schulze has announced his own version of the Proto-Lezgian reconstruction ([8], [9], [10]). The amount of Schulze’s etymologies for individual Lezgian roots that has already been published by the author is insufficient for any final decisions, but I must note that a significant number of Schulze’s diachronic ideas does not look acceptable from my point of view. For example, Schulze treats inherited Udi *muχ* ‘fingernail’ as a Persian loanword (actually Persian *mex* means 'nail, peg', not ‘nail, fingernail’ and therefore it cannot be a source of borrowing, whereas the Udi word has nice etymological cognates in other Lezgian languages); the Udi verb for ‘to sit’ is analyzed by Schulze as an old compound of two verbal roots, ‘to come’ and ‘to sit down’, with the semantic explanation ‘to sit’ < *‘he came and sat down’ that is typologically improbable; and so on.

The second option of marking cognation is a formal algorithm, based on **phonetic similarity**. There are two most popular approaches to the automatic establishing of cognate word pairs between the given wordlists: Levenshtein distances and consonant classes. In fact, the method of consonant classes may be considered a crude variation on the measurement of Levenshtein distances. Below I will rely on consonant classes (I am not aware of any publications which compare the discussed approaches and demonstrate that consonant classes yield significantly less reliable results than Levenshtein distances).

The method of consonant classes was proposed by A. Dolgopolsky in 1964 ([11], English version: [12]) and successfully tested by various authors on the data of various languages of Eurasia (e.g., [13], [14], [15], [16], [17]).

This method implies that the phonetic alphabet used in our studies can be divided into several non-intersecting subsets (classes) so that phonetic mutations between the sounds of one class during natural language development are typologically more normal than mutations between sounds that belong to different classes. Typology of sound changes is not sufficiently advanced yet (but cf. [18], [19] for progress in this area), therefore such a division can only be based on the intuition and experience of individual linguists. Below, I operate with classes currently accepted in the *Global Lexicostatistical Database* project (GLD) [2]: http://starling.rinet.ru/new100/sound.pdf [Accessed 16.02.2014]. The system of transcription is normally adapted to the unified transcription system of GLD, which is mostly based on the IPA alphabet: http://starling.rinet.ru/new100/UTS.htm [Accessed 16.02.2014]. The GLD classes run as follows:

P-class (labials): p b ɓ β f v …
T-class (dentals): t d ɗ θ ð …
S-class (front affricates & fricatives): c ʒ č ǯ s z š ž …
Y-class (palatal glides): y …
W-class (labial glides): w ʍ …
M-class (labial nasals): m ɱ …
N-class (non-labial nasals): n ɳ ɲ ŋ …
Q-class (lateral affricates): ƛ ᴌ …
R-class (liquida): r ɾ l ɬ ɭ ɫ …
K-class (velars & uvulars): k g x ɣ q χ ʁ …
zero-class or H-class: ħ ʕ ʜ ʢ ʡ h ɦ ʔ and any vowels.

Using this simplified transcription system (*P T S Y W M N Q R K H*) we can code any real wordforms or morphemes included into comparison. Note that elements of the zero-class and such features as coarticulation, prosody, phonation are deleted from the structure. Vocalic or laryngeal onsets and vocalic or laryngeal finals, however, are coded as *H*. Thus both hypothetical forms *tasa* and *dʰüʒo* are coded as *TSH*; *alaq* and *ʡärx* = *HRK*; *na* and *ŋoʔ* = *NH*; *pkʰot* and *baqʼaθ* = *PKT*; *wahat* and *ʍad* = *WT*. Non-initial *Y* and *W* (weak glides) are treated as *H*, thus *ka*, *kay*, *kawa* = *KH*, whereas *kat* and *kayat* = *KT*.

As follows from the above, two forms from compared languages possessing identical simplified transcriptions have a better chance to appear to be etymological cognates than forms whose simplified transcriptions differ.

All the Lezgian wordlists have been coded in this way, whereupon cognations have been automatically established in the Starling software: two forms are marked as cognates if the first two consonants in their simplified transcriptions coincide. For instance, the words for ‘ashes’: Kryts *räq* (*RK*) = Aghul *rüqːʸ* (*RK*) ≠ Tsakhur *yiqˤ-* (*YK*) ≠ Archi *diqʼːˤ-* (*TK*), even though in reality all the forms originate from one proto-root (i.e., from the same ancestral root of the proto-language). On the contrary, Udi *kul* ‘hand’ (*KL*) = Tsakhur *χɨlʸ* ‘id.’ (*KL*), even though these forms actually originate from different proto-roots.

For tree rooting, the 110-item wordlist of the Chechen literary language [20] has been introduced into comparison as an outgroup. Chechen was chosen as a language genetically related to Lezgian within the North Caucasian linguistic family (or more narrowly within its Nakh-Dagestanian cluster), on the one hand, and as a lect which is definitely not a member of the Lezgian group on the other. Etymological comparison between Chechen and Lezgian is based on [5] with some corrections from [20].

## Methods

Lexicostatistical trees were produced by several phylogenetic methods.

1. Modified neighbor joining method, designed by S. Starostin for lexicostatistical analysis and implemented in the Starling software (method Starling neighbor joining, hence StarlingNJ); see [21]. StarlingNJ is an agglomerative hierarchical clustering method which can be called “stepwise bottom-up distance averaging”. The distance between two lects *A* and *B* is 1 minus the percentage of shared Swadesh items, e.g., if there are 87 coinciding slots between the 100-item wordlists of *A* and *B*, dist(A, B) = 0.13. If a non-modern lect is involved, its percentage is automatically adapted to AD 1950 according to the accepted molecular clock model [21], [22].

At the first step, the nearest two taxa *A* and *B* are combined into a higher-level taxon (AB). The distance between (AB) and another taxon *x* is computed by the following rule:

IF dist(A, x) > 0.25 OR dist(B, x) > 0.25:

$$\text{dist}(\text{AB},\;\text{x})=\frac{\text{dist}(\text{A},\;\text{x})\;+\;\text{dist}(\text{B},\;\text{x})}{2}$$

ELSE:

dist(AB,x)=max(dist(A,x),dist(B,x))

The “ELSE” case represents a special adjustment of the averaging formula for secondary contacts between closely related lects. At the next step, this operation is repeated. If the nearest taxa are (AB) and *C*, the distance between ((AB)C) and another taxon *x* is (when regular averaging was applied):

$$\text{dist}((\text{AB})\text{C},\;\text{x})=\frac{\text{dist}(\text{AB},\;\text{x})\;+\;\text{dist}(\text{C},\;\text{x})}{2}=\frac{\text{dist}(\text{A},\;\text{x})}{4}+\frac{\text{dist}(\text{B},\;\text{x})}{4}+\frac{\text{dist}(\text{C},\;\text{x})}{2}$$

Note that dist((AB), C) ≠ dist(A, (BC)).

When the same Swadesh slot is occupied by more than 1 word (i.e., by several synonyms), all possible pairs of involved words between two languages are compared within this slot: if there is at least one matching pair, the whole slot is treated as a match.

The StarlingNJ trees were produced in the Starling software v.2.5.3 (see [23], [21]) from the lexicostatistical database which represents a multistate matrix with synonymy allowed. For node dating, the so-called “experimental method” was applied, according to which each Swadesh item possesses an individual relative index of stability [24], [25]. The non-parametric bootstrap test was performed (10 000 pseudoreplicates). The hierarchical agglomerative clustering produces by its very definition a rooted tree. Dates of the nodes were established by strict molecular clocks, see [26], [27], [22], [28] on scale calibration and further details. For data elaborated by the StarlingNJ method, two kinds of trees are offered: a tree with binary nodes only (as produced by the NJ algorithm), and the same tree, where neighboring nodes are joined in one node if the temporal distance between them is 300 years or less (300 years correspond to mutation of *ca*. 1.5 words in a lect, a reasonable calculation error). The trees were visualized in Starling and then manually redrawn for best appearance.

1. Standard neighbor joining method (hence NJ), see [29], [30]. The trees were produced in the SplitsTree4 software v.4.13.1 [31] from the binary lexicostatistical matrix (NEXUS format) which was generated from the original multistate matrix by coding the presence (“1”) or absence (“0”) of each proto-root in each of the 21 languages (Swadesh items superseded by loanwords or simply not documented are marked as “?”). Total 484 characters (proto-roots) for etymology-based calculations and 678 characters for phonetic similarity-based calculations. The non-parametric bootstrap test was performed (10 000 pseudoreplicates). The trees were rooted by the outgroup (the Chechen wordlist). The trees are not dated. The trees were visualized in the FigTree software (v.1.4.0). Also additional trees were produced by the BioNJ method [32], these appeared to be topologically identical to the NJ ones.
2. Unweighted pair group method with arithmetic mean method (hence UPGMA), see [33], [30]. The trees were produced in the SplitsTree4 software v.4.13.1 from the binary matrix described above. The non-parametric bootstrap test was performed (10 000 pseudoreplicates). The trees were rooted by the outgroup (the Chechen wordlist). The trees are not dated. The trees were visualized in the FigTree software (v.1.4.0).
3. Markov chain Monte Carlo method under Bayesian framework (hence Bayesian MCMC), see [30], as it was for the first time applied to linguistic data in [34]. The trees were produced in the MrBayes software v.3.2.1 [35] from the binary matrix described above. I used F81 model with rates = gamma. The program was run 4 times using 4 concurrent Markov chains; the Chechen language was marked as an outgroup. Each run produced 5 000 000 tree generations with samples taken every 500 generations. For each run, first 25% tree generations were discarded as a burn-in. The consensus trees were rooted by the outgroup (the Chechen wordlist). The trees are not dated. The trees were visualized in the FigTree software (v.1.4.0).
4. Unweighted maximum parsimony method (hence UMP), see [30]. The trees were produced in the TNT software (Willi Hennig Society edition of TNT, v.1.1, May 2014, see [36]) from the binary matrix described above by the branch-and-bound (“Implicit enumeration”) algorithm. Obligatory binarization of nodes was prohibited (“Collapse trees after the search”); the Chechen language was marked as an outgroup. For the etymology-based wordlist, 4 optimal trees of equal cost were obtained and the strict consensus tree was produced, for which the non-parametric bootstrap test was performed (1000 pseudoreplicates). For the phonetic similarity-based wordlist, 1 optimal tree was obtained and the same bootstrap test was performed. The trees were rooted by the outgroup (the Chechen wordlist). The trees are not dated. The trees were visualized in the FigTree software (v.1.4.0).

## Results: Etymology-Based Trees

**Fig 2:**
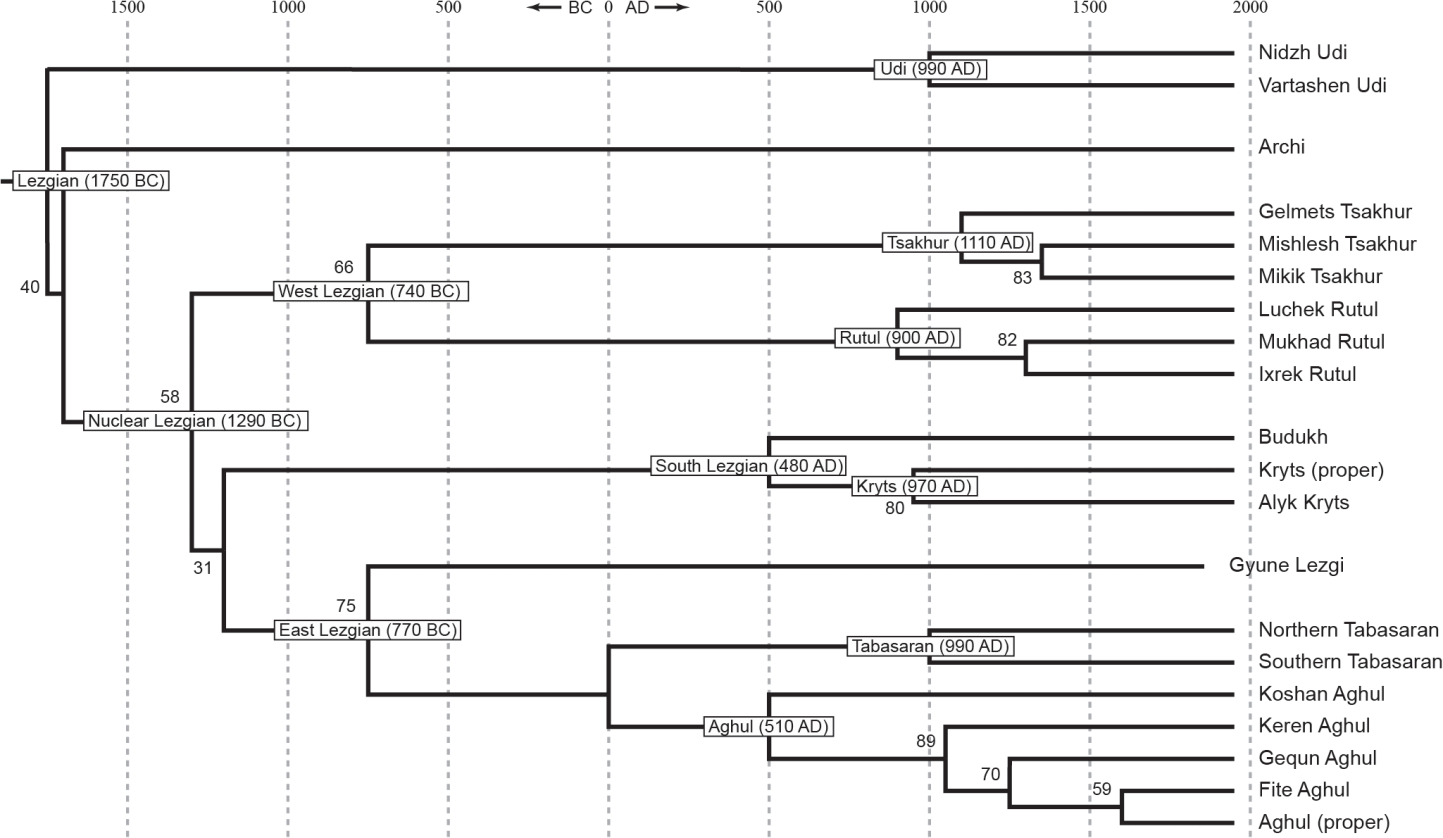
Etymology-based phylogenetic tree of the Lezgian lects produced by the StarlingNJ method from the multistate matrix (binary nodes only). Bootstrap values are shown near the nodes (not shown for stable nodes with bootstrap value ≥ 95%). The tree is dated.

**Fig 3:**
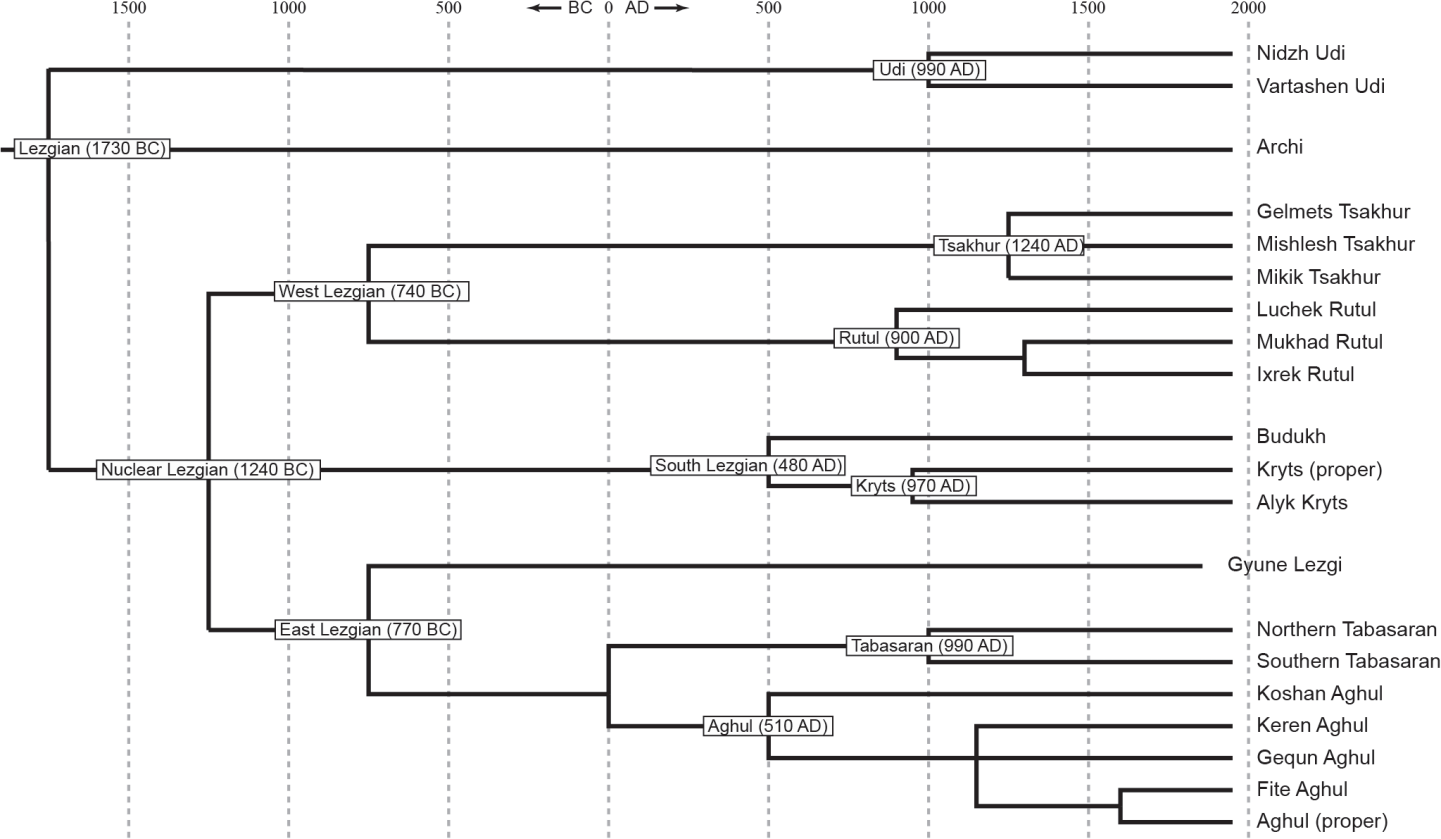
Etymology-based phylogenetic tree of the Lezgian lects produced by the StarlingNJ method from the multistate matrix (neighboring nodes are joined if the distance between them is 300 years or less). The tree is dated.

**Fig 4:**
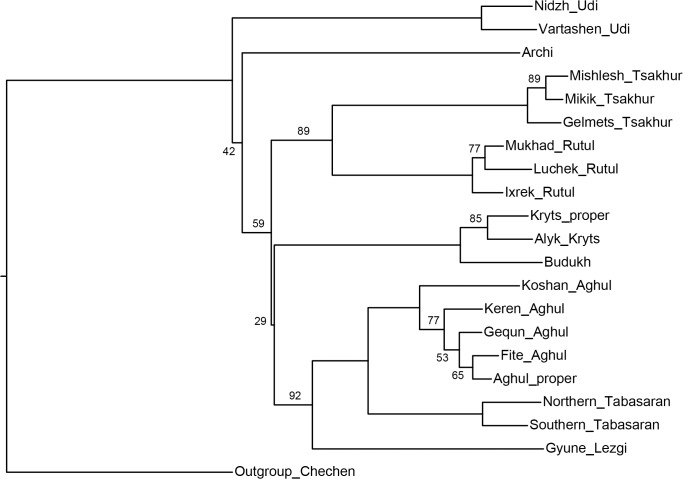
Etymology-based phylogenetic tree of the Lezgian lects produced by the NJ method from the binary matrix in the SplitsTree4 software. Bootstrap values are shown near the nodes (not shown for stable nodes with bootstrap value ≥ 95%). Branch length reflects the relative rate of cognate replacement as suggested by SplitsTree4. The BioNJ method yields the same topology.

**Fig 5:**
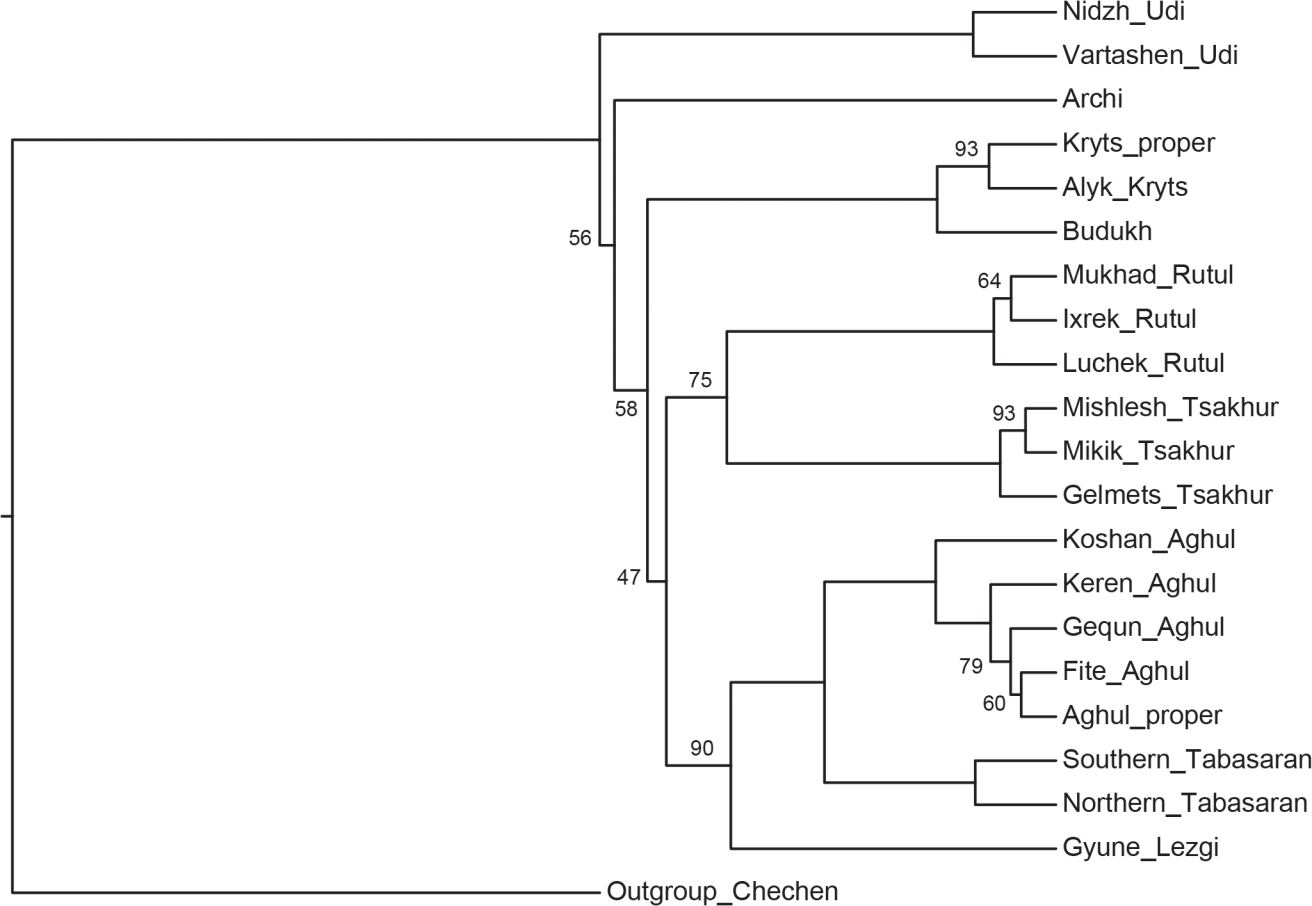
Etymology-based phylogenetic tree of the Lezgian lects produced by the UPGMA method from the binary matrix in the SplitsTree4 software. Bootstrap values are shown near the nodes (not shown for stable nodes with bootstrap value ≥ 95%). Branch length reflects the relative rate of cognate replacement as suggested by SplitsTree4.

**Fig 6:**
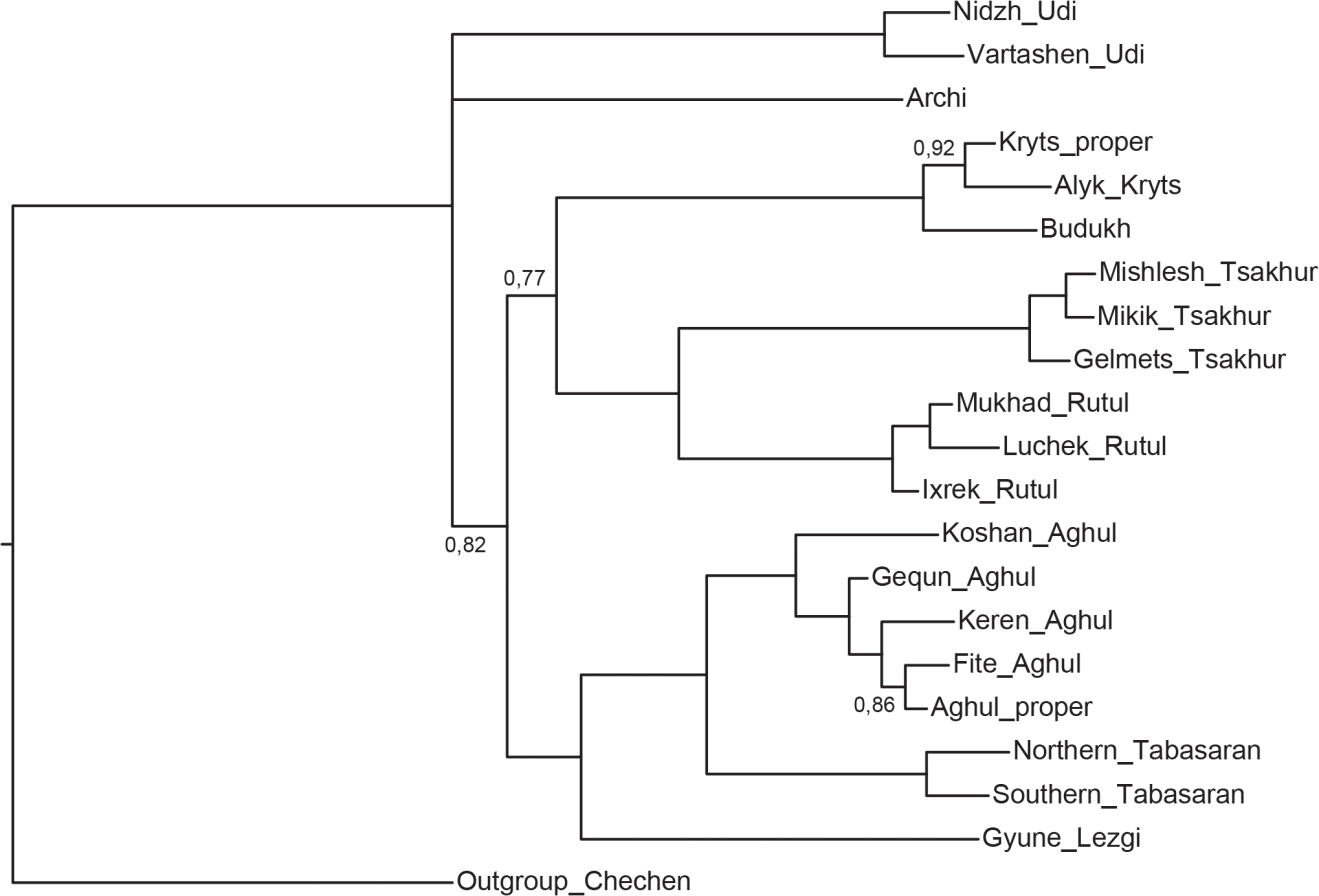
Etymology-based consensus phylogenetic tree of the Lezgian lects produced by the Bayesian MCMC method from the binary matrix in the MrBayes software. Bayesian posterior probabilities are shown near the branches (not shown for stable branches with *P* ≥ 0.95). Branch length reflects the relative rate of cognate replacement as suggested by MrBayes.

**Fig 7:**
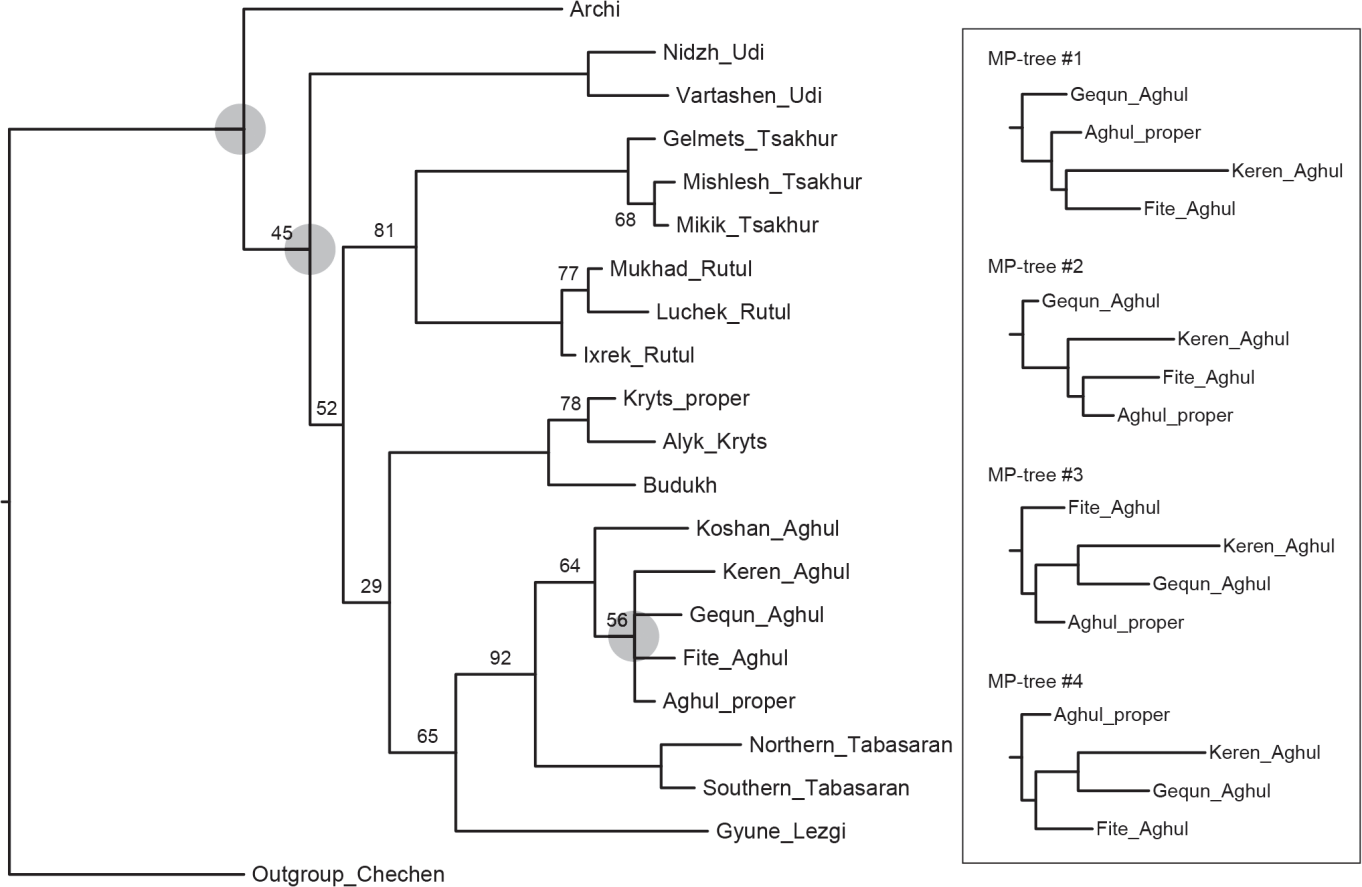
Etymology-based consensus phylogenetic tree of the Lezgian lects produced by the UMP method from the binary matrix in the TNT software. Bootstrap values are shown near the nodes (not shown for stable nodes with bootstrap value ≥ 95%). Branch length reflects the relative rate of cognate replacement as suggested by TNT. The four optimal trees only differ in the Aghul node as shown in the above panel. Nodes which appeared to be problem as compared to other phylogenetic methods are shadowed.

**Fig 8:**
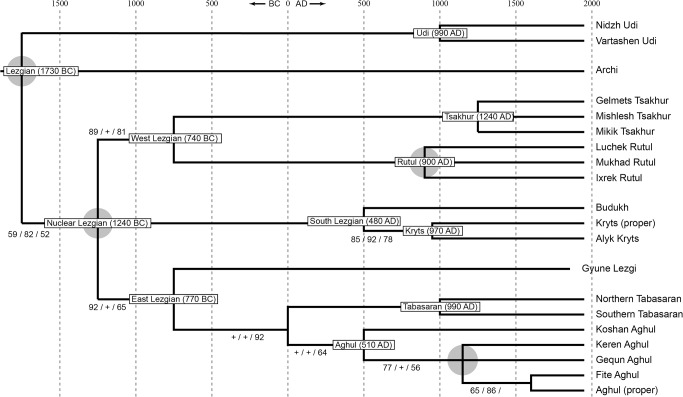
Manually constructed consensus etymology-based phylogenetic tree of the Lezgian lects based on the StarlingNJ, NJ, BioNJ, UPGMA, Bayesian MCMC, UMP methods. The gray ellipses mark 4 joined nodes which cover binary branchings that differ depending on the method. Probability values are shown in the following sequence: NJ / MCMC / UMP (“+” means that P ≥ 0.95 in an individual method; not shown for nodes with P ≥ 0.95 in all methods). StarlingNJ dates are proposed.

The following trees with etymological cognations were obtained:

- Fig. 2, StarlingNJ method with binary nodes only;
- Fig. 3, StarlingNJ method with neighboring nodes joined;
- Fig. 4, NJ method;
- Fig. 5, UPGMA method.
- Fig. 6, Bayesian MCMC method.
- Fig. 7, UMP method.
- Fig. 8, manually constructed consensus tree.

The difference between the obtained trees does not seem substantial (especially if the UMP-tree is excluded). Let us examine them.

1. All distance-based methods, i.e., StarlingNJ, NJ, BioNJ, UPGMA (Figs. 2, 4, 5) suggest consecutive bifurcations with the Udi branch split off first and the rest divided into the Archi and Nuclear Lezgian (a.k.a. Samur) branches. The distance between two nodes (the separation of Udi and the separation of Archi) is, however, minimal in all the distance-based trees, as follows from the tree visualization and the probabilistic values of the branches, and under the assumption of the temporal error of 300 years in StarlingNJ (Fig. 3) the first split of the Lezgian group appears to be a three-way one: Udi, Archi, Nuclear Lezgian. On the contrary, the character-based Bayesian method MCMC (Fig. 6) immediately suggests the ternary split into Udi, Archi and Nuclear Lezgian. It should be remembered that the distance-based methods (StarlingNJ, NJ, BioNJ, UPGMA) are only able to produce binary trees. As for the UMP-tree, this one seriously differs from the others, see below.
2. All the methods suggest the three-part division of the Nuclear Lezgian sub-group: (1) proto-West Lezgian [Tsakhur, Rutul], (2) proto-South Lezgian [Kryts, Budukh], (3) proto-East Lezgian [Aghul, Tabasaran, Lezgi]. The difference is found out in the hierarchy of the splits. The StarlingNJ and NJ (Figs. 2, 4) as well as UMP (Fig. 7) methods suggest that West Lezgian splits off first and then South Lezgian and East Lezgian bifurcate. The UPGMA method (Fig. 5) suggests that South Lezgian splits off first. Finally, the Bayesian MCMC method (Fig. 6) suggests that East Lezgian splits off first. The distance between two nodes (i.e., consecutive bifurcations between West, South and East proto-languages) is, however, minimal in all the trees, as follows from the tree visualization and the probabilistic values of the branches, and under the assumption of the temporal error of 300 years in StarlingNJ (Fig. 3) the split of the Nuclear Lezgian sub-group appears to be a three-way one: West, South and East.
3. The Aghul dialects. All the methods reconstruct the initial separation of the Koshan dialect and the distinct Proper Aghul/Fite clade (that meets intuitive expectations), but then begin to contradict each other. The distance-based methods, i.e., StarlingNJ, NJ, BioNJ, UPGMA (Figs. 2, 4, 5) suggest the consecutive separation of the Keren dialect and then of the Gequn one, whereas the character-based Bayesian MCMC method (Fig. 6) reconstructs the reverse order: initially Gequn, then Keren. The distance between two nodes (i.e., consecutive bifurcations between Keren, Gequn and Proper Aghul/Fite) is, however, minimal in all the trees, as follows from the tree visualization, and under the assumption of the temporal error of 300 years in StarlingNJ (Fig. 3) the split of the proto-Aghul language after the separation of Koshan appears to be a three-way one: Keren, Gequn and Proper Aghul/Fite. As for the UMP-tree, this one differs from the others, see below.
4. Apparently the most serious discrepancy between the obtained trees (except for the UMP one) concerns the topology of three Rutul dialects. The StarlingNJ and UPGMA methods (Figs. 2, 5) suggest that the Luchek dialect splits off first. On the contrary, NJ and Bayesian MCMC (Figs. 4, 6) as well as UMP (Fig. 7) reconstruct the initial separation of Ixrek. At the same time, in Fig. 2 (StarlingNJ), two Rutul nodes are chronologically remote enough not to get joined under the assumption of the temporal error of 300 years (Fig. 3). As follows from the tables of distances, for both the multistate and binary matrices, the lexicostatistical distances in the Rutul part of the tree are not ultrametric, but far from it (Tables 1, 2). Such a situation is not normal for contemporary taxa under the assumption of the constant or nearly constant rate of cognate replacement within the Swadesh wordlist, and individual phylogenetic methods suggest different solutions. From the linguistic point of view, the Rutul data can be explained by two perturbing factors: (1) interdialectal loans and contact-driven homoplasy (there is no way to reveal such cases at the current stage of research); (2) imperfection of the available lexicographic sources which do not permit to compile Swadesh wordlists more accurately. It is difficult to say which one of the two topologies more adequately conforms to the historical reality (cf. [37]), but in any case quantitative methods of genealogical classification can hardly be fully applicable to a situation of mutually intelligible contacting lects (the dialect continuum), as we see in the Rutul territory.
5. Finally, the UMP method appears to be somewhat isolated among other methods. This one suggests a tree which is partially incompatible with other obtained trees as well as with our informal intuitive ideas about the Lezgian phylogeny (see Fig. 7, where the problem nodes are shadowed). Firstly, the main shortcoming is that the Archi language appears to be the first outlier, whereas Udi which is formally the second outlier actually tends to be joined with the next Nuclear Lezgian node. Secondly, the topology of the Aghul dialects remains unresolved, Proper Aghul/Fite are not posited as a robust distinct clade. Since the UMP method contradicts both other phylogenetic methods and traditional expert Lezgian classification (see Sec. 4), I suppose that UMP results should be used with caution.

**Table 1:** Reverse lexicostatistical distances for 3 Rutul dialects (higher percentage of the shared basic vocabulary meaning greater closeness): multistate input matrix.

	Ixrek	Luchek
Mukhad	0.96	0.94
Ixrek	—	0.91

**Table 2:** Reverse lexicostatistical distances for 3 Rutul dialects (higher percentage of the shared basic vocabulary meaning greater closeness): binary input matrix.

	Ixrek	Luchek
Mukhad	0.973	0.969
Ixrek	—	0.955

Taking into account the aforementioned discrepancies, the following consensus phylogenetic tree of the Lezgian lects can be manually constructed, see Fig. 8. In this tree, the neighboring nodes are joined, (1) if the temporal distance between them is ≤ 300 years as calculated by the StarlingNJ method, see Figs. 2–3; or (2) if their topology depends on the individual phylogenetic methods (the only exception is the Proper Aghul/Fite Aghul clade which is missing from the UMP-tree, but present in all other methods and in traditional expert classifications, and hence included into the consensus tree). The gray ellipses mark 4 joined ternary nodes which cover binary branchings that differ depending on the method: three of them are automatically obtained under the assumption of the temporal error of 300 years, whereas the fourth one joins the Rutul dialects discussed above. As one can see, the topology of the consensus tree (Fig. 8) is identical to the StarlingNJ-tree (Fig. 3) except for the additional joining of the Rutul dialects into one ternary node. As noted in the following section, the consensus tree (Fig. 8) coincides with the traditional expert classification of that group and thus can be used as the “gold standard”.

## Previously Proposed Classifications

The manually constructed consensus etymology-based tree of the Lezgian group (Fig. 8) with two outliers (Udi and Archi) and a large group of nuclear a.k.a. Samur lects (which divide into three clusters: West, South and East, in the latter, Lezgi splits off first) conforms to the following previously proposed phylogenetic classifications.

1. The traditional expert classification, see, e.g., [38] with further references. The obtained phylogeny also fulfils the traditional views on dialect phylogeny of individual languages. Out of three involved Tsakhur dialects, Mishlesh and Mikik are specifically close to each other, whereas Gelmets is an outlier [39], [40], [41]). All the tested methods suggest the same binary branching, although the Tsakhur dialects are indeed joined in one ternary node in the consensus tree (Fig. 8) due to short temporal distance between the original binary nodes. As for three involved Rutul dialects (Mukhad, Ixrek, Luchek), there is no strict classification of these: Mukhad and Ixrek are indeed detached from each other, but Luchek is a “mixed” or “transitional” dialect as described in [37]. In accordance with it, phylogenetic methods propose different branching patterns for the Rutul clade (see above). Out of five involved Aghul dialects (Koshan, Keren, Gequn, Aghul proper, Fite), Koshan is traditionally regarded as the most detached one, whereas Aghul proper and Fite are specifically close to each other [42], [43]. The same is suggested by all the tested methods: Koshan is an outlier, Aghul proper and Fite form a distinct clade. The position of Keren and Gequn within the non-Koshan clade is not determined by Caucasologists and in accordance with it, phylogenetic methods propose different branching patterns for Keren and Gequn. It is important to point out that although Caucasologists generally agree about the outlines of the classification, a fully worked out comparative study with identification of shared innovations is not available (but cf. [44] for progress in this area).
2. Some previous, rougher, lexicostatistical calculations [44], [45] which were based on the etymologized 100-item wordlists, elaborated by a UPGMA-like method. Note that there is an unfortunate misprint in the Lezgian phylogenetic tree, offered in [45]: Archi is joined to the Nuclear Lezgian node. Actually Archi is a second outlier which splits off after Udi, but prior to Nuclear Lezgian, as follows from the reverse lexicostatistical distance matrix, Table 2 in [45].
3. The formal classification of the *Automated Similarity Judgment Program* project [46], based on the non-etymologized 40-item wordlists. The average Levenshtein distances between individual wordforms with the same meaning yield the distance matrix between languages which is further elaborated by the neighbor joining algorithm (bootstrap tests are not applied). Note that in [46], the Khinalugh language is also included into the Lezgian group that is apparently incorrect.

On the contrary, the lexicostatistical classification in [1] (100-item wordlists, etymologized and elaborated by the StarlingNJ method in the Starling software), according to which Archi is the fourth Nuclear Lezgian branch, is not confirmed and should now be rejected; since the author has not disclosed the actual lexical dataset used to produce the tree, his results remain unverifiable.

Similarly, Schulze’s opinion ([47], [10]; but contra [48]) that Udi-Caucasian Albanian belongs to the East Lezgian cluster of the Nuclear subgroup, together with Aghul, Tabasaran and Lezgi, appears to be incorrect. Schulze published his own version of the Caucasian Albanian and Udi Swadesh wordlists, accompanied with several grammatical features, and compared these to general Lezgian data [10]. Unfortunately, Schulze did not provide any explanations for his specific version of formal phylogeny (strictly speaking, it even remains unclear which forms within the offered Lezgian wordlist are treated by the author as etymological cognates and which ones as etymologically unrelated items), and at the same time the general lexicographic quality of the compiled wordlists is rather low (especially it concerns the Caucasian Albanian data: for example, the Caucasian Albanian word which actually means ‘kin, clan’ is quoted for the Swadesh meaning ‘seed (botanic)’; ‘month’—for the Swadesh meaning ‘moon’; ‘clay’—for the Swadesh meaning ‘earth’; and so forth). For these reasons, I have to conclude that Schulze failed to present any formal arguments in support of his tree. On an intuitive basis, Schulze’s classification seems just as wrong—I am not aware of any specialists in Lezgian and generally Caucasian studies that would regard Schulze’s Lezgian tree as acceptable.

## Additional Test: Phonetic Similarity-Based Trees

Automatical language comparison, based on phonetical similarity, is a suitable tool for a quick language relationship check and tentative phylogeny reconstruction. When a great number of languages is involved, whose historical phonology is not sufficiently advanced, such an automated preliminary lexicostatistics serves as a good basis for further more detailed studies on that language group [25].

Rate of phonetic changes is not constant, but varies in a very wide range. It is a normal situation, when sound changes are modest and gradual during centuries and then get abrupt and massive. For example, such Semitic languages as Epigraphic South Arabian or Jibbali demonstrate almost bi-unique correspondence with the reconstructed Proto-Semitic consonants [49], it implies that Epigraphic South Arabian and Jibbali words retained their original consonant “skeletons” practically untouched during several millennia. These conservative languages contrast, e.g., with Modern English which underwent heavy phonetic mutations as compared with Proto-Indo-European or at least with Proto-Germanic (partially it is highlighted by English orthography).

An instance of substantially different rate of phonetic changes within the same clade is the Italic language group. French and Italian evolved from Vulgar Latin in the mid-1^st^ millennium AD, i.e., *ca*. 1,500 years separate modern French and Italian from their common proto-language. Despite close relationship, French and Italian vary in respect of historical sound changes: Italian is relatively conservative, whereas French is rather innovative. It is illustrated in Table 3 which contains ten French-Italian lexical pairs from the beginning of the Swadesh wordlist accompanied with their Latin protoforms. As one can see, in all cases excepting ‘fingernail’, Italian forms are phonetically closer to Latin than the French ones are.

**Table 3:** Ten etymological matches between the French and Italian Swadesh wordlists plus the Latin ancestral forms.

	French	Italian	Latin
‘all’	tu	tutːo	totus
‘ashes’	sɑ̃dʁ	čener-i	kiner-is
‘big’	gʁɑ̃	grande	grandis
‘bite’	mɔʁdʁ	mɔrdere	mordere
‘black’	nwaʁ	nero	niger
‘blood’	sɑ̃	saŋgwe	sangwis
‘bone’	ɔs	ɔsːo	osːum
‘fingernail’	ɔ̃gl	uŋgya	ungula
‘cold’	fʁwa	fredːo	friːgidus
‘dog’	šyɛ̃	kane	kanis

In other words, phonetic changes in natural language evolution represent a stochastic process which can hardly be described by evolutionary models. As concerns the specific method of consonant classes adopted in the present paper, it should be noted that in language history, the bulk of inferred phonetic shifts is typologically trivial (by definition), i.e., the shifts happen within the limits of phonetically justified consonant classes. But certainly mutations between different consonant classes are also rather common and almost inevitable. The ratio of trivial mutations to non-trivial ones is random for individual cases.

If an algorithm of marking cognation is based on phonetic similarity, some etymologically related words are marked as non-cognate, whereas some phonetically similar, but etymologically unrelated words are marked as cognate. The material difference between the Lezgian etymology-based multistate matrix and the phonetic similarity-based one can be illustrated with a pair of distantly related lects (Archi + Budukh) and a pair of closely related lects (Koshan Aghul + Northern Tabasaran). Archi + Budukh: 81 common Swadesh slots; out of them, 38 (47%) are etymologically related, only 17 (21%) are phonetically similar. Koshan Aghul + Northern Tabasaran: 95 common Swadesh slots; out of them, 70 slots (74%) are etymologically related, only 49 slots (52%) are phonetically similar.

As follows from the above, phonetic similarity-based cognation marking adds noise into the input matrix as compared with the etymology-based approach. Since Lezgian languages generally demonstrate a lot of non-trivial phonetic changes, it is interesting how various methods cope with phylogeny reconstruction based on the noisy dataset. Below their results are compared with the consensus etymology-based tree (Fig. 8) which coincides with the traditional expert classification.

**Fig 9:**
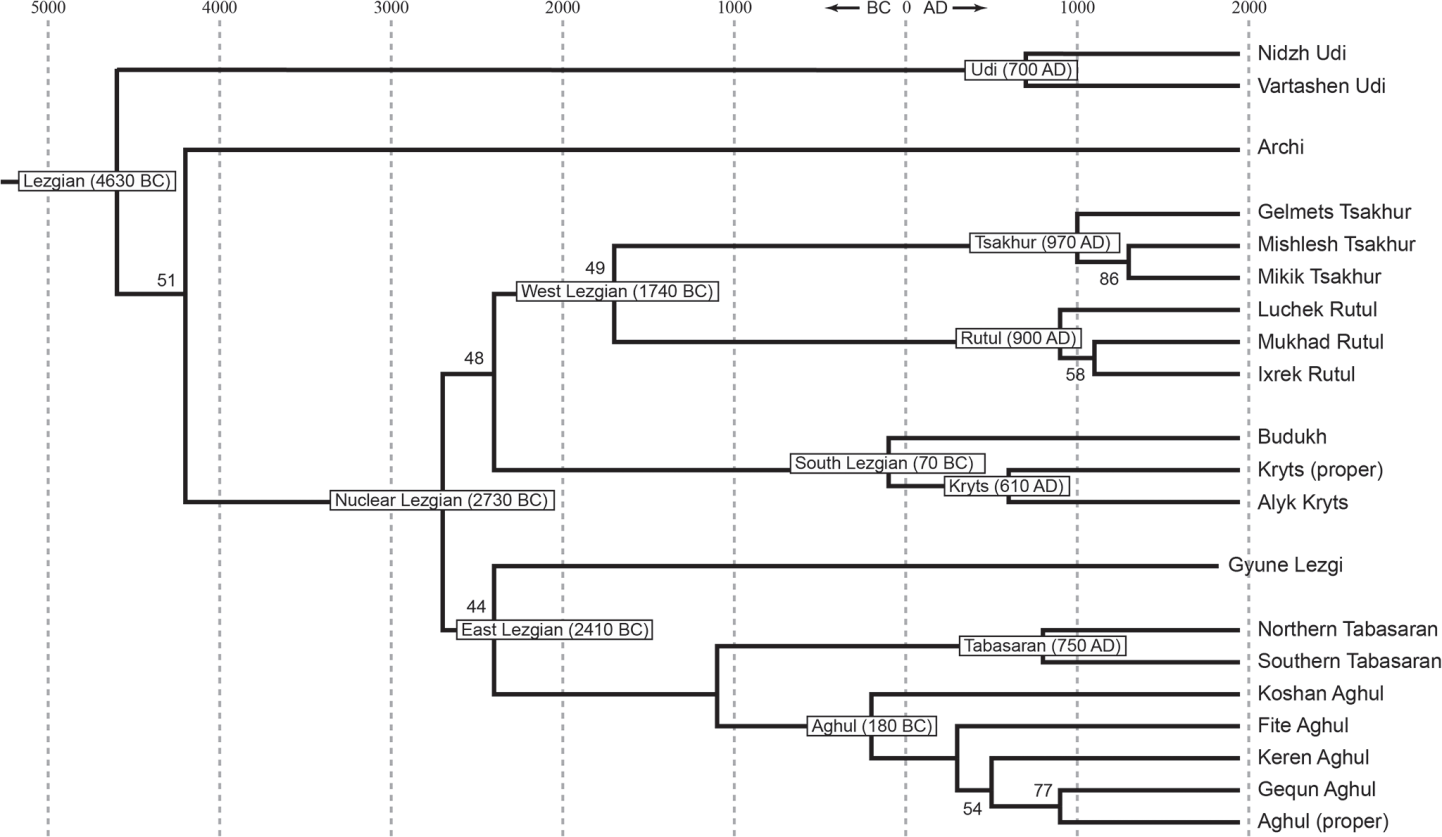
Phonetic similarity-based phylogenetic tree of the Lezgian lects produced by the StarlingNJ method from the multistate matrix (binary nodes only). Bootstrap values are shown near the nodes (not shown for stable nodes with bootstrap value ≥ 95%). The tree is dated.

**Fig 10:**
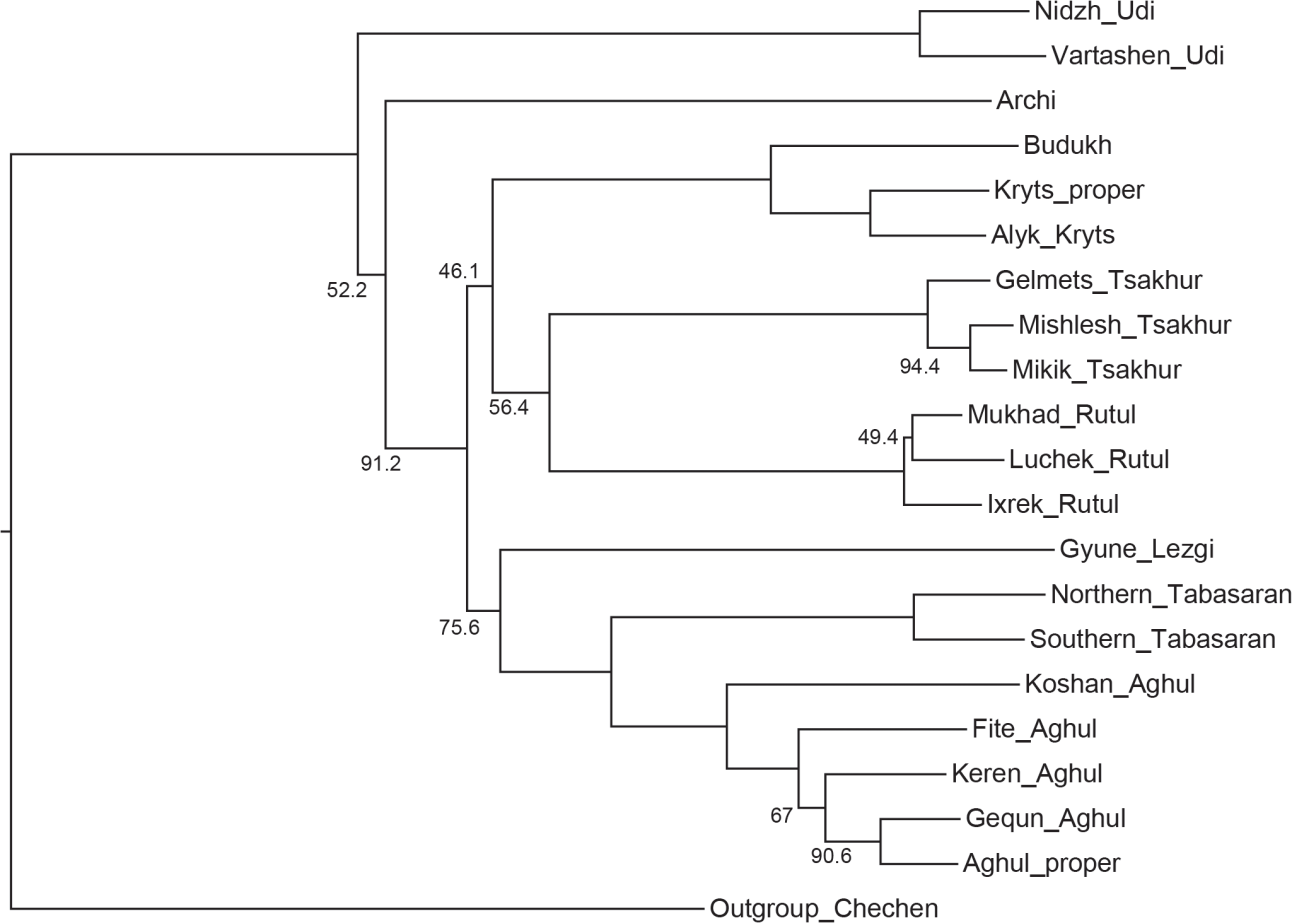
Phonetic similarity-based phylogenetic tree of the Lezgian lects produced by the NJ method from the binary matrix in the SplitsTree4 software. Bootstrap values are shown near the nodes (not shown for stable nodes with bootstrap value ≥ 95%). Branch length reflects the relative rate of cognate replacement as suggested by SplitsTree4. The BioNJ method yields the same topology.

**Fig 11:**
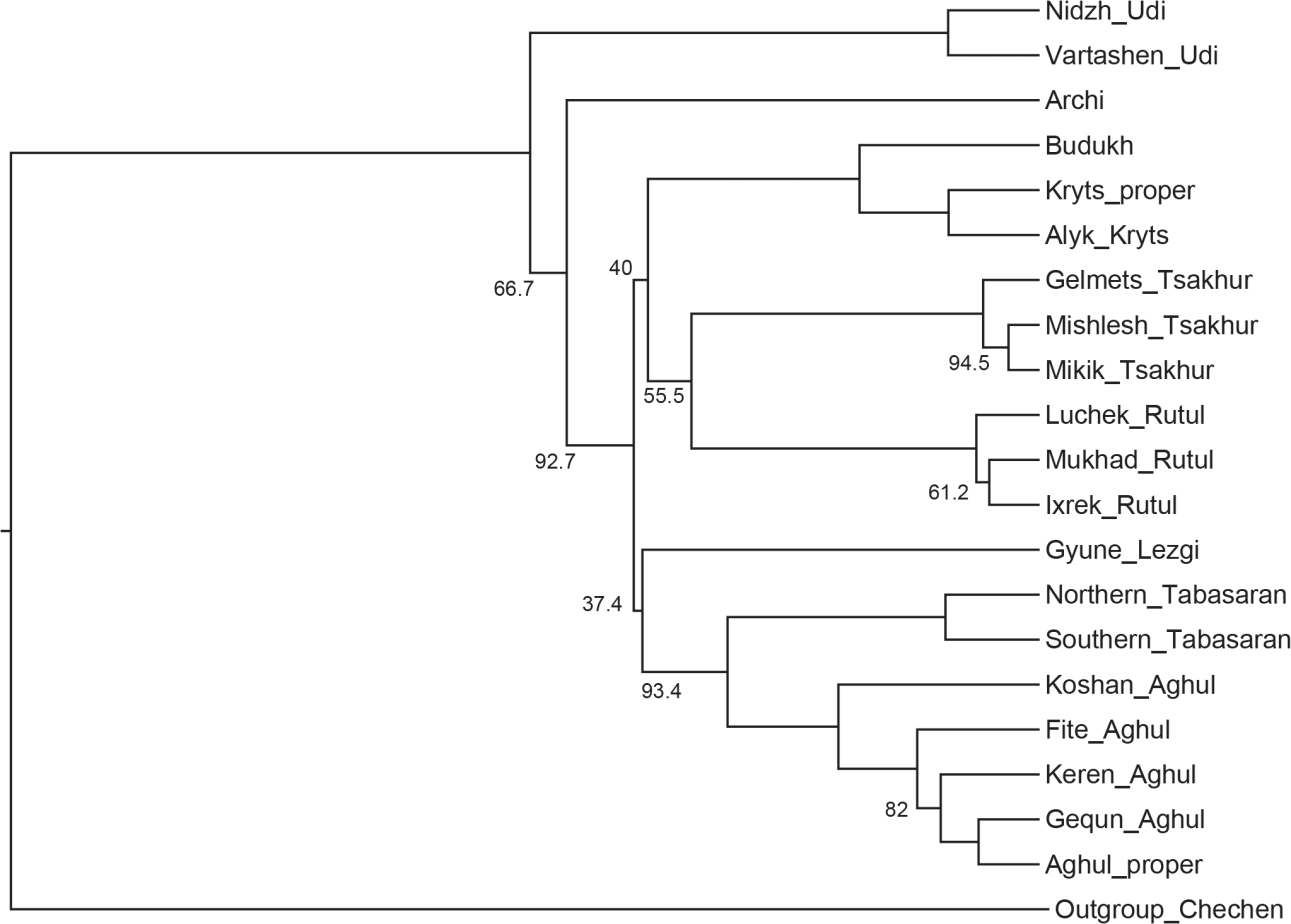
Phonetic similarity-based phylogenetic tree of the Lezgian lects produced by the UPGMA method from the binary matrix in the SplitsTree4 software. Bootstrap values are shown near the nodes (not shown for stable nodes with bootstrap value ≥ 95%). Branch length reflects the relative rate of cognate replacement as suggested by SplitsTree4.

**Fig 12:**
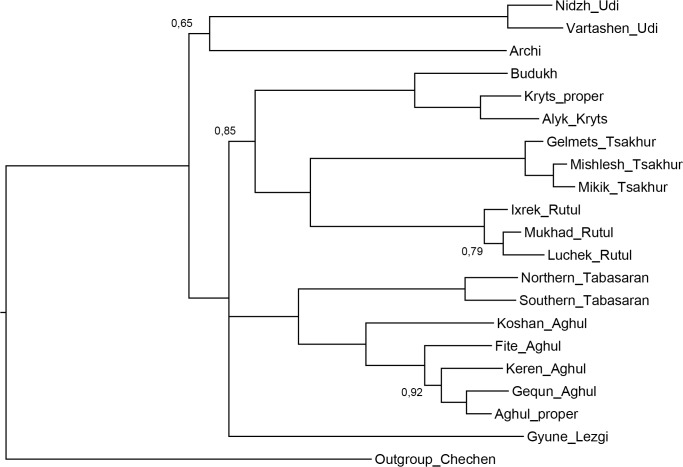
Phonetic similarity-based consensus phylogenetic tree of the Lezgian lects produced by the Bayesian MCMC method from the binary matrix in the MrBayes software. Bayesian posterior probabilities are shown above the branches (not shown for stable branches with *P* ≥ 0.95). Branch length reflects the relative rate of cognate replacement as suggested by MrBayes.

**Fig 13:**
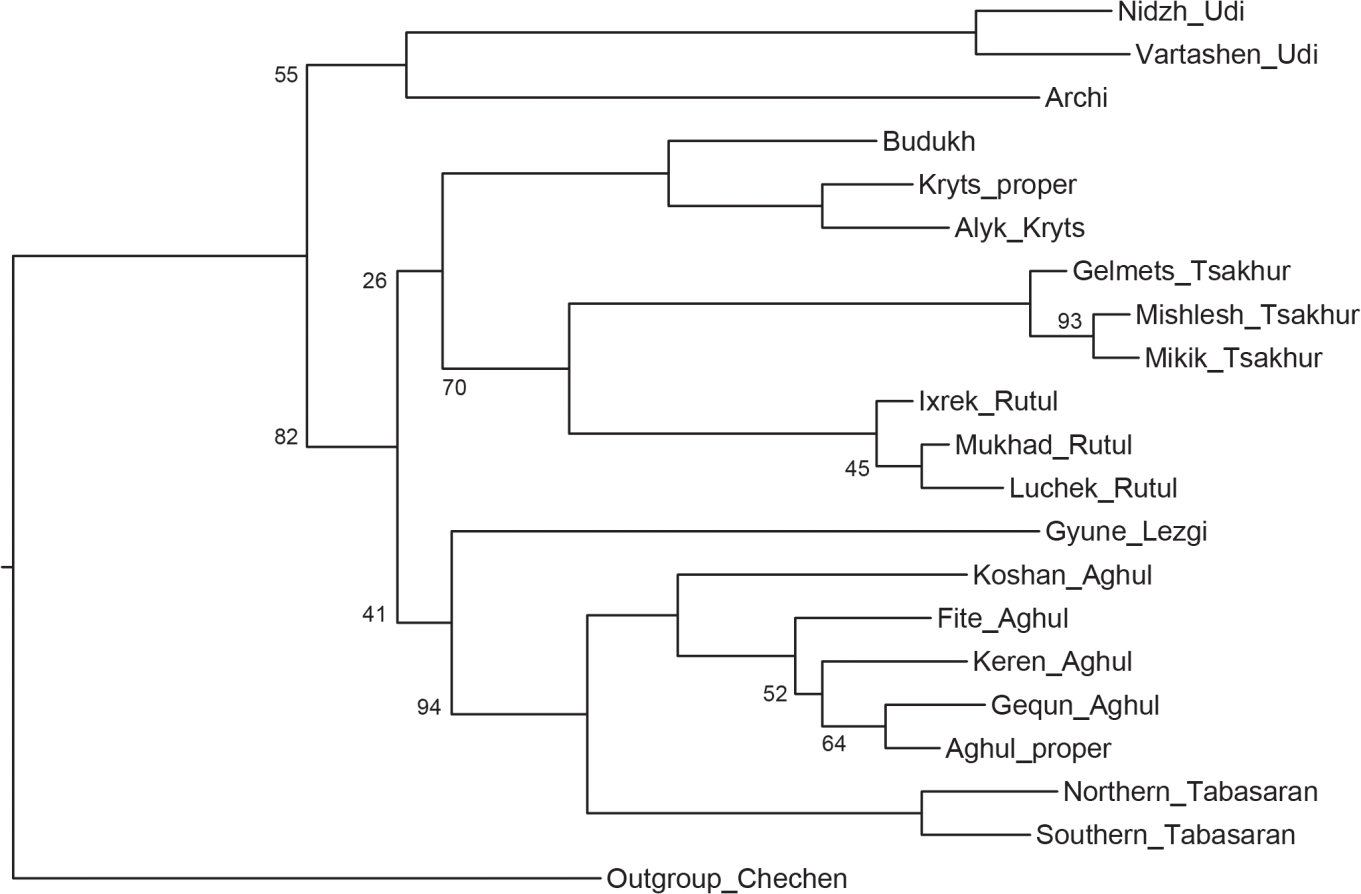
Phonetic similarity-based phylogenetic tree of the Lezgian lects produced by the UMP method from the binary matrix in the TNT software. Bootstrap values are shown near the nodes (not shown for stable nodes with bootstrap value ≥ 95%). Branch length reflects the relative rate of cognate replacement as suggested by TNT.

The following Lezgian trees with phonetic similarity-based cognations were obtained:

- Fig. 9, StarlingNJ method with binary nodes only;
- Fig. 10, NJ method;
- Fig. 11, UPGMA method.
- Fig. 12, Bayesian MCMC method.
- Fig. 13, UMP method.

As one can see, the distance-based methods—StarlingNJ, NJ, BioNJ, UPGMA (Figs. 9–11)—produce trees which are rather similar to the consensus etymology-based tree (Fig. 8). The StarlingNJ tree (Fig. 9) topologically coincides with the UPGMA tree (Fig. 11), whereas the NJ and BioNJ trees (Fig. 10) differs in the Rutul dialects—the same discrepancy as in the case of the etymology-based wordlist. In sum, the main flaws of the distance-based methods as compared with the consensus etymology-based tree are the following ones.

1. Topology of the Aghul dialects is incorrect (Fite and Aghul proper are disjoined).
2. The split of proto-Nuclear Lezgian tends to appear to be a four-way one, not a three-way one: (1) South, (2) West, (3) Lezgi, (4) Tabasaran-Aghul. I.e., the Lezgi language rather looks like a separate branch within the Nuclear Lezgian subgroup.

Thus, for the phonetic similarity-based matrix, I consider the results of the distance-based methods as good.

On the contrary, the character-based methods appear to be less reliable. The main flaws of the UMP tree (Fig. 13) are the following ones.

1. Two outliers, Udi and Archi, form a distinct clade.
2. As in the case of the distance methods (Figs. 9–11), UMP suggests the unlikely topology of the Aghul dialects (Fite and Aghul proper are disjoined).

The result of the Bayesian MCMC (Fig. 12) method is even worse.

1. Two outliers, Udi and Archi, form a distinct clade.
2. The split of Proto-Nuclear Lezgian is a three-way one, but the structure of the taxa is unexpected: (1) South & West, (2) Tabasaran & Aghul, (3) Lezgi.
3. As in the case of the distance methods (Figs. 9–11), Bayesian MCMC suggests the unlikely topology of the Aghul dialects (Fite and Aghul proper are disjoined).

## Conclusions

As noted above, the Lezgian 110-item database [3] has several important features.

1. The database consists of a relatively large amount of taxa: 20 lects. Among them, there are both languages which existed isolated for a long time, e.g., Archi, and languages which actively contact with other languages of the same group (that potentially induces a jump of lexicostatistical matches due to contact-driven retentions and contact-driven homoplasy), e.g., Aghul.
2. It is not a serious overstatement to say that there is a consensus about the Lezgian phylogeny among Caucasologists (the outliers Udi and Archi plus the Nuclear Lezgian sub-group with three clusters: West, South, East with further dialectal classification, see Sec. 4);
3. The Lezgian group can probably be characterized as medium or slightly above the average among the world’s language groups with regard to reliability and particularity of the available lexicographic descriptions.
4. The general quality, that is, the “purification efficiency” of the Lezgian lexicostatistical lists (as well as other wordlists published in the *Global Lexicostatistical Database* project [2]) is unprecedentedly high for world linguistics.

It makes the Lezgian database a good testing area for appraisal of phylogenetic methods.

In the theoretical paper [50], the adequacy of the main phylogenetic methods is tested by simulation of various linguistic situations. The authors conclude that the most reliable method is maximal parsimony (MP), followed by Bayesian MCMC, then by NJ, whereas UPGMA appears to be substantially less accurate then the others.

There are, however, specific difficulties in application of the MP method with binary characters to linguistic data. One of the reasons for that can be the unconformity of the used model to our ideas of natural language evolution. The MP method depends on homoplasy (i.e., parallel or back developments) to a greater extent than other methods. Correspondingly, for minimization of the effect of homoplastic disturbances, the authors of [50] propose to use individual costs of characters (Weighted maximum parsimony), assigning higher costs to those characters which do not demonstrate homoplasy in the given linguistic data. In the case of binary characters with equal cost of change between the states, homoplasy is equivalent to the presence of two incompatible characters in the input matrix, that is, two characters which take all four possible pairs of states: “00”, “01”, “10”, “11” (see, e.g., [51]). The MP method, tested in [50], treats changes between the states “0” and “1” as equal. But if we—as in the present paper—use a binary matrix, where “1” denote a marked state of the character and “0”—an unmarked one (e.g., “1” = presence, whereas “0” = absence of the specific proto-root with the specific Swadesh meaning in the given language), so the change 1 > 0 (loss of the root) is not a significant event, it can occur independently in different languages, and such a loss may hardly be regarded homoplastic. Thereby to detect *linguistic* homoplasy, it is needed to reconstruct ancestral character states that is actually a non-trivial theoretical and practical task [52], particularly the reconstruction is impossible without the established phylogenetic tree—as a result we get in a vicious circle.

Laying aside this disputable aspect of Barbançon et al.’s paper, it can be seen that the authors give preference to the character-based method (MP, Bayesian MCMC) over the distance-based ones (NJ, UPGMA), and this is the main conclusion of the paper [50] (similar views on hierarchy of reliability of phylogenetic methods are gradually prevailing in modern molecular biology). As a quantitative assessment, it is proposed in [50] that all the tested phylogenetic methods, except for UPGMA, reconstruct *ca*. 90% of the branches of the true tree.

For the etymology-based input matrix, experiments with the Lezgian lexicostatistical database present, however, a more comforting picture, if one believes that each branch of the true tree has been reconstructed at least by one of the tested methods except for UMP (i.e., each branch of the true tree is reproduced in Figs. 2–6.

Under the assumption of a relatively small temporal error (with the joining of neighboring nodes within such a time span, see Fig. 8 with comments), it can be seen that the tested StarlingNJ, NJ, UPGMA, Bayesian MCMC methods only interfere with each other in the hierarchy of the three Rutul dialects. The consensus etymology-based tree (Fig. 8) comprises 33 branches plus 1 additional branch, if the Rutul dialects are not joined into a ternary node: total 34 branches. The discrepancy between the methods in the topology of the Rutul dialects yields an error in 1 branch of 34, that is, all the methods (except for UMP) have correctly reconstructed from 97% to 100% of the true branches. An unexpected result of the Lezgian test is the relatively low plausibility of the obtained UMP-tree (Fig. 7) that straightly contradicts the theoretical calculations of [50].

As for the phonetic similarity-based matrix, the distance methods (StarlingNJ, NJ, UPGMA) appear to be more resistant to such inaccurate and irregular input data than the character-based methods (Bayesian MCMC, UMP). It is a somewhat unexpected result. More tests of this kind are needed to clarify the situation.

The examined Lezgian data support some propositions which serve as an ideological basis of the *Global Lexicostatistical Database* project.

1. Lexicon is a reliable tool for language classification. It is sometimes claimed by linguists that grammar is preferable for phylogenetic purposes (see. e.g., [53]), but I argue that grammatical—phonetic, morphological, syntactical—characters should be used for language phylogeny with caution (see Sec. 4 on Schulze’s unsuccessful attempts to revise Lezgian phylogeny with the mixed set of lexical and grammatical characters; the most well-known experiment of that kind is probably [54], where several phylogenetic methods are tested on the Indo-European data with the mixed lexico-grammatical matrix). First of all, proposed grammatical features are mostly binary, which means the probability of chance coincidences is high (cf., e.g., definitely unrelated Modern English and Thai which share a lot of stable and valuable grammatical characters such as “order of subject, object and verb”, “intensifiers and reflexive pronouns”, “order of relative clause and noun”, and so on [55]). Secondly, specific grammatical characters may not be universal (when we design a specific list of grammatical characters for an individual language family, it can make our results biased). Thirdly, they can readily produce common areal isoglosses (especially if we are dealing with lects whose relationship is still understood by the speakers), whereas in most cases, it is difficult to detect the source of such an innovation. Fourthly, grammatical characters form a system that means a change of one character may entail a change of other characters. On the contrary, such shortcomings are characteristic of lexical data to a considerably lesser degree (for example, the majority of loanwords and the direction of borrowing can be revealed proceeding from our knowledge of historical phonetics and morphology of the compared language.).
2. If the involved languages are etymologically elaborated and historical phonetics of this language group is well studied, i.e., the sound correspondences are established, the reliability of a phylogenetic tree depends in the first place not on a calculation method, but on general quality and “purification efficiency” of the input data. In other words, not on computer work, but on work of a linguist which laboriously surveys individual lects (although some phylogenetic methods such as maximal parsimony can indeed give rise to some doubt). See Sec. 3 for detail.
3. If the historical phonetics of the involved languages is not described and we are forced to mark cognation by simple and formal algorithms, based on phonetic similarity, then the distance-based methods can be even more reliable than the character-based ones. See Sec. 5 for detail.

## Supporting Information

S1 File Multistate matrix in MS Excel format with etymology-based cognations (incl. the wordlists). (XLSX)

S2 File Binary matrix in NEXUS format with etymology-based cognations. (NEX)

S3 File Reverse distance matrix, generated from the multistate matrix (etymology-based cognations) in the Starling software. (XLSX)

S4 File Distance matrix, generated from the binary matrix (etymology-based cognations) in the SplitsTree4 software. (TXT)

S5 File Multistate matrix in MS Excel format with phonetic similarity-based cognations. (XLSX)

S6 File Binary matrix in NEXUS format with phonetic similarity-based cognations. (NEX)

S7 File Reverse distance matrix, generated from the multistate matrix (phonetic similarity-based cognations) in the Starling software. (XLSX)

S8 File Distance matrix, generated from the binary matrix (phonetic similarity-based cognations) in the SplitsTree4 software. (TXT)

S9 File Map of the modern Lezgian lects (Fig. 1) in vector format. (EPS)
